# Anger, Cynical Distrust, Nightmare Distress and Insomnia Among Nursing Personnel

**DOI:** 10.3390/jcm15103837

**Published:** 2026-05-15

**Authors:** Athanasios Tselebis, Argyro Pachi, Christos Sikaras, Dimitrios Kasimis, Evgenia Kavourgia, Ioannis Ilias

**Affiliations:** 1 Psychiatric Department, Sotiria Thoracic Diseases Hospital of Athens, 11527 Athens, Greece; kasimisdimitrios@yahoo.com (D.K.); ekavourgia@gmail.com (E.K.); 2 Nursing Department, Sotiria Thoracic Diseases Hospital of Athens, 11527 Athens, Greece; cris.sikaras@gmail.com; 3 Department of Endocrinology, Hippokration General Hospital of Athens, 11527 Athens, Greece; iiliasmd@yahoo.com

**Keywords:** anger, cynical distrust, hostility, nightmare distress, insomnia, sleep disorders, nursing staff, occupational mental health, serial mediation

## Abstract

**Background**: The nursing profession is recognized as a high-risk occupation, with the emotional toll on healthcare workers reaching a critical point. A complex interplay of anger and cynicism, often stemming from systemic pressures and chronic moral injury, seems to increasingly affect nurses’ professional and personal lives. This psychological strain does not end when the shift ends; rather, it often manifests as insomnia and nightmare distress, creating a vicious cycle of exhaustion and emotional instability. This article explores how anger, cynical distrust, nightmare distress and insomnia are interrelated and jeopardize the well-being of nursing staff and what these “invisible” symptoms reveal about the current state of healthcare by confirming their prevalence rates. **Methods**: This cross-sectional study was conducted online in October 2025 and included 441 hospital nurses who completed the Dimensions of Anger Reactions-5 (DAR-5), the 8-item Cynical Distrust scale (CDS-8), the Nightmare Distress Questionnaire (NDQ) and the Athens Insomnia Scale (AIS). **Results**: The prevalence rates of anger, nightmare distress and insomnia were 41.5%, 6.6%, and 62.1%, respectively. Based on the CDS-8 scores, a notable proportion (20.9%) of nurses fell within the highest quartile of CDS-8 scores (CDS-8 > 29), indicating relatively elevated cynical distrust within this sample; this threshold is sample-derived and does not correspond to a validated clinical cut-off. Hierarchical multiple regression analysis indicated that the DAR-5 explained 22.1% of the variance in AIS, while an additional 10.2% was explained by NDQ and another 1.5% by the CDS-8. Both cynical distrust and nightmare distress displayed a chain mediation pattern in the association between anger and insomnia; however, given the cross-sectional design, the temporal order of these variables cannot be confirmed. **Conclusions**: Anger exhibited significant direct and indirect associations with insomnia, with cynical distrust and nightmare distress acting as serial mediators in this cross-sectional model. Findings from this cross-sectional study tentatively suggest that future intervention efforts targeting insomnia in nurses might benefit from addressing anger alongside nightmare distress and cynical attitudes; however, experimental studies are needed to confirm whether such interventions would be effective.

## 1. Introduction

The mental health of healthcare professionals is a critical area of research in current scientific literature, since it is directly linked to both the quality of care provided and the overall functioning of healthcare systems [1,2]. Nursing staff, as a cornerstone of healthcare systems, are constantly exposed to intense emotional demands, heavy workloads, and frequent ethical dilemmas, which increase the likelihood of psychological difficulties. In this context, concepts such as anger, cynical distrust, nightmare distress, and insomnia emerge as significant factors affecting nurses’ well-being and functioning [3,4].

Contemporary research studies evidence rising anger issues among nurses driven by a combination of systemic factors and failures, moral injury and distress, workplace violence and aggression, lack of support and respect, along with physical and emotional exhaustion [5,6]. The healthcare workplace is one of the most emotionally challenging environments because it involves caring for people when suffering and nurses are at the forefront of care delivery. Anger usually serves as a defense mechanism to disguise more vulnerable emotions like anxiety, fear, and frustration, feelings of powerlessness, perceived injustice, betrayal, guilt, a sense of threat, and a loss of control [7]. The accumulation of anger in highly demanding work environments can reinforce negative interpretations of social interactions, thereby increasing cynical distrust. In other words, individuals with elevated levels of anger are more likely to attribute hostile motives to others, reinforcing a vicious cycle of emotional and cognitive dysfunction [8,9].

Unaddressed anger may have severe repercussions for both nurses and patients, impacting nurses’ physical and mental health and threatening patients’ safety and care standards [10]. Instead of safely expressing their concerns about the reasons that justify angry emotions, nurses usually feel they are obliged to suppress these negative emotions and remain silent due to fear of retaliation [11]. Persistent suppressed anger among nurses may lead the way to cynicism that significantly erodes the work environment and jeopardizes organizational stability [12,13].

Cynicism has been viewed as an aspect of hostility in the sense of distrust of the motives of others, and in a broader context, it is associated with negative assessments of other people’s intentions and a tendency to doubt the honesty or integrity of individuals [14]. Studies on cynicism have evidenced associations with negative physical and mental health outcomes [15,16]. It has been noted that individuals with high levels of cynicism experience increased levels of stress, isolation and hopelessness, and a reduced likelihood of pursuing emotional or practical sources of support [17,18]. Furthermore, individuals with high levels of cynicism, primarily due to their reduced cognitive flexibility, tend to exhibit a diminished ability to adapt [19]. One of the most significant consequences of cynicism among nurses is that it leads to a sense of futility and isolation, where nurses feel alienated from their colleagues and disillusioned regarding the mission of the organization [20].

More specifically, the concept of cynical distrust has its origins in hostility theories and refers to a relatively stable cognitive schema characterized by a generalized suspicion regarding the motives of others and the absolute conviction that others act primarily in a selfish and/or malicious manner [21,22]. Although this concept has been extensively studied in the field of health, particularly in relation to cardiovascular diseases [23], it remains relatively unexplored in the nursing profession.

In the context of the nursing profession, cynical distrust appears to be closely related to the concept of cynicism, as outlined in the theory of professional burnout. According to the model by Maslach and Leiter (2016), cynicism is a core dimension of burnout and manifests as emotional detachment and a negative attitude toward patients and work [24]. However, cynical distrust differs in that it places greater emphasis on the cognitive dimension of distrust and the negative evaluation of others’ intentions, a feature that can have a more profound impact on interpersonal relationships in the workplace [22,25].

Furthermore, cynical distrust can be viewed as an extension of organizational cynicism, which encompasses elements of disillusionment, distrust, and negative feelings toward the organization [26]. In highly stressful environments, such as hospitals, where professionals often experience a lack of support and recognition, the emergence of such attitudes is particularly plausible. Chronic exposure to stressful situations can reinforce cognitive schemas of distrust, leading to an increased cynical attitude [27].

To clarify the distinction between these three related but distinct constructs, namely, “cynicism,” “cynical distrust,” and “organizational cynicism”, it should be noted that cynicism is conceptualized as a broad dispositional and attitudinal tendency; cynical distrust (as operationalized by the CDS-8) is defined as a stable cognitive schema of interpersonal suspicion with origins in hostility theory; and organizational cynicism as a context-specific disillusionment directed toward the individual’s workplace. It should be emphasized that this study focuses specifically on cynical distrust in the hostility/health psychology tradition.

Nurses with high levels of cynical distrust typically experience a gloomy worldview with inherent emotions of resentment and suspicion, further entrenching a pessimistic perspective. This constantly hostile mindset increases the likelihood of experiencing nightmares by creating a high-stress environment that fosters their recurrence [28,29]. A state of hypervigilance combined with a negative cognitive appraisal style and/or obsessive negative thinking, along with the physical and emotional exhaustion that discourages adaptive coping strategies, social isolation and lack of support all contribute to the emergence of nightmares [30,31,32]. The high levels of emotion and fear after waking, i.e., nightmare distress, is because the emotional exhaustion and reduced cognitive flexibility associated with high cynicism make nightmares more threatening and lead to high levels of distress. In this way, cynical distrust can serve as a predisposing factor for nightmare distress.

Neurocognitive models suggest that nightmare distress is associated with difficulties in emotional processing and regulation [33,34]. The presence of intense negative emotions, such as anger, combined with cognitive schemas of distrust, may intensify the content and frequency of nightmares, as well as their emotional impact [35]. At the same time, cynical distrust and anger appear to be linked to sleep disorders, such as insomnia. Cognitive hyperarousal, which characterizes individuals with high levels of distrust and hostility, has been identified as a key mechanism in the development and maintenance of insomnia [36]. Negative thoughts and hypervigilance can make it difficult to initiate and maintain sleep, while also affecting its quality.

The relationship between insomnia and nightmares is bidirectional; each condition can trigger or aggravate the other [37]. Frequent or recurrent nightmares are a direct cause of insomnia symptoms, either because people deliberately delay going to sleep and resist falling asleep to avoid the distressing content of their dreams, or because they have difficulty falling back asleep after being abruptly awakened by a nightmare, or even because, over time, sleeping has become a source of anxiety instead of rest [38]. Either way, nightmares predominantly occur during rapid eye movement (REM) sleep, leading to frequent nocturnal awakenings that disrupt the natural progression and restorative benefits of sleep, causing insomnia. Nurses are considered a high-risk group for both insomnia and nightmares, with prevalence rates exceeding those found in the general adult population [4,39].

The relationship between anger and insomnia among nurses has already been studied [3,40,41]. Also, research studies evidence the strong positive association between anger and cynicism [42,43]. The relationship between insomnia and cynicism among nurses is frequently explored in the context of occupational burnout, conceptualized as part of the depersonalization dimension [24,44,45]. Research investigating the relationship between nightmare distress and cynical distrust [46] especially among nurses is scarce [47,48] and is, in conjunction with anger and insomnia and their interrelations, to our knowledge very limited in the existing literature. Among nursing staff, these factors may interact in a unique way, considering the challenging working conditions, such as exposure to traumatic events, rotating shifts, and the emotional involvement with patients. Despite the importance of these factors, the combined study of cynical distrust, anger, and sleep disorders remains limited in the scientific literature.

Therefore, in this study, we aim to evaluate the levels of and explore the relationships between anger, cynical distrust, nightmare distress and insomnia among Greek hospital nurses. Furthermore, a review of the literature revealed no previous studies investigating the chain mediating associations of cynical distrust and nightmare distress on the relationship between anger and insomnia. The theoretical framework of this study was based on the hypothesis that anger would be associated with insomnia both directly and indirectly through the associational sequence of cynical distrust and nightmare distress. Specifically, we argue that: (a) anger, as a trait-like disposition, predisposes individuals to develop stable negative cognitive schemas, including cynical distrust, through repeated hostile appraisal processes; (b) chronic cynical distrust, characterized by hypervigilance and negative cognitive appraisals, creates a persistent threat-based affective state that is known to be a predisposing factor for nightmare distress; and (c) nightmare distress then directly disrupts sleep continuity, contributing to insomnia symptoms. Also, it should be acknowledged that this ordering is theoretically proposed and empirically testable only through longitudinal or experimental designs.

Therefore, the above hypothetical model seeks to elucidate the underlying associational sequence through which anger, cynical distrust, nightmare distress and insomnia interact in order to gain a substantive understanding of the hypothetical psychological process that undermines the well-being of nurses and to enable exploratory suggestions for intervention efforts targeting insomnia that warrant future experimental testing. To achieve this goal, we formulated the following hypotheses:

**Hypothesis** **1.** *Anger is positively associated with and predicts insomnia.*

**Hypothesis** **2.** *Cynical distrust mediates the relationship between anger and insomnia.*

**Hypothesis** **3.** *Nightmare distress mediates the relationship between anger and insomnia.*

**Hypothesis** **4.** *Cynical distrust and nightmare distress play a chain mediating role in the relationship between anger and insomnia.*

## 2. Materials and Methods

### 2.1. Research Design

To achieve the aforementioned goals, we used a homogeneous convenience sampling method in order to conduct this cross-sectional study. Nurses with at least one year of professional experience who worked in Greek hospitals were recruited. Google Forms was used to gather the data, and the email addresses obtained from Greek nurses’ scientific and professional registries were utilized to distribute the online survey. An anonymous link to the Google Forms online research platform was included in the invitation email that participants received. By selecting the “I agree” option on the first page of the online survey, consenting participants indicated that they agreed to participate voluntarily and this was regarded as informed consent. Nurses who agreed to participate in this study and subsequently completed the remaining online questionnaire sections made up the study’s sample. Hospital nurses who were not on duty for any reason were excluded.

### 2.2. Study Participants

The research was carried out in October of 2025. The sample size was determined using Cochran’s formula [49]. A minimum sample size of 385 participants was needed given the target population of 27,103 people [50], a 95% confidence level (i.e., the z score was 1.96), a 5% confidence interval (i.e., the margin of error for the proportion being estimated was 0.05), and an assumption of a 50% response rate (i.e., the population proportion was 0.5). 441 people responded to the 500 invitations that were sent out via email (response rate: 88%). This high response rate likely reflects the targeted nature of the invitation and the professional motivation of participants.

### 2.3. Minimal Sample Size Calculation

A post hoc power analysis was performed using G*Power (version 3.1.9.7) for the multiple linear regression model. With a total sample size of 441 and seven predictors, the achieved power to detect a medium effect size (f^2^ = 0.15) at a significance level of α = 0.05 was >0.99 for both the overall model and the individual predictors. These results confirm that the study was sufficiently powered to identify statistically significant associations. Also, a Monte Carlo formal statistical power analysis for indirect effects, tailored for the structural mediation model, is provided in the Results Section.

### 2.4. Ethical Considerations

The World Medical Association Declaration of Helsinki (1975, revised 2008), the General Data Protection Regulation (GDPR-2016/679) of the European Union, and the International Committee of Medical Journal Editors’ guidelines were all followed when conducting this study. The Ethics Committee of Clinical Research at the General Hospital for Thoracic Diseases of Athens “SOTIRIA” approved the study protocol (Approval Number: 31984/25 November 2024).

### 2.5. Measurement Tools

Respondents were asked to state demographic and professional information, such as their gender, age, and years of work experience, after providing their consent and before completing the following series of questionnaires:

#### 2.5.1. Dimensions of Anger Reactions-5 (DAR-5)

Anger is measured using the Greek version of the DAR-5 [51,52,53], which is a brief five-item scale. On a 5-point Likert scale, where 1 represents never or almost never and 5 corresponds to always or almost always, respondents rate their anger frequency, intensity, duration, aggression, and its interference with social relations, over the past four weeks. After adding up the five scores, a total score between 5 and 25 is obtained. Higher scores denote a more intense experience of anger, whereas lower scores indicate a less intense experience. For the scale, ≥12 is the cut-off point. Cronbach’s alpha for the internal reliability of the scale used in this study was 0.811.

#### 2.5.2. Eight-Item “Cynical Distrust” Scale (CDS)

Cynicism was measured using the Cynical Distrust Scale (CDS). The eight items on the Greek version of the scale [54] are rated on a 5-point Likert scale, with 1 denoting “strongly disagree” to 5 denoting “strongly agree”. A high value of cynical distrust is indicated by a high sum of the eight items. An indicative average value for the Greek population as a whole is 25.5 [54]. With a Cronbach’s alpha of 0.814, the study’s questionnaire had very good internal reliability.

#### 2.5.3. The Nightmare Distress Questionnaire (NDQ)

This is the most popular questionnaire for assessing nightmare-related distress [4,55,56,57]. This 13-item Greek version questionnaire covers a wide range of topics about nightmares. These items are rated on a 5-point response set with higher scores indicating higher levels of distress. While items like “Do nightmares interfere with the quality of your sleep?” are rated from 1 = not at all to 5 = a great deal, most questions like “Do you have difficulties coping with nightmares?” range from 1 = never to 5 = always. The final question on interest in nightmare therapy is coded from 1 (not at all interested) to 5 (very interested). This questionnaire has a total score between 13 and 65 and scores higher than 39 are deemed abnormal. With Cronbach’s alpha values ranging from α = 0.83 to α = 0.88, the NDQ has shown excellent internal consistency [57]. In this study, Cronbach’s alpha was determined to be α = 0.91, indicating high internal consistency.

#### 2.5.4. Athens Insomnia Scale (AIS)

Based on the diagnostic criteria established by the 10th Revision of the International Classification of Diseases and Related Health Problems (ICD-10), the Athens Insomnia Scale (AIS) is a self-report measurement tool designed to assess the severity of insomnia (i.e., how much specific sleep difficulties have affected the responders during the past month). Eight items make up the scale, with the first five measuring nighttime sleep (sleep induction, nighttime awakenings, final awakening, total sleep duration, and overall sleep quality) and the last three items evaluating daytime dysfunction (drowsiness throughout the day, functioning, and well-being). Each item has a response score between 0 and 3, and the total score ranges from 0 to 24. Higher scores indicate more severe insomnia. The presence of insomnia is indicated by a diagnostic threshold of six [58]. Good psychometric qualities have been shown for the Greek version of the AIS [59], and in this study, the Cronbach’s alpha coefficient was measured at α = 0.878.

Discriminant validity between AIS and NDQ scales was established with the heterotrait-monotrait (HTMT) ratio, which is a comparison of the correlations between indicators across different constructs with the correlations of indicators within the same constructs. In this study, the value of 0.811 is below the threshold value of 0.85, thus confirming discriminant validity of the constructs.

### 2.6. Statistical Analysis

First, the common method bias was examined using the Harman single-factor test because self-report questionnaires were used to collect the data. To find the percentage of variance explained by the first largest factor, this method employs exploratory factor analysis, in which all variables are loaded on a single factor and restricted, so that there is no rotation. Descriptive statistical methods were utilized to estimate means and standard deviations for continuous variables and to determine the percentage of respondents who scored higher than the cutoff values of clinically significant study variables. To assess the representativeness of the sample, we also used t-tests and *χ*^2^ tests to compare the sample’s years of professional experience, age, and gender to the overall Greek nursing population. To compare continuous variables as to gender, independent t-tests were performed. Hedges’ g-value from the independent samples t-test results was used to calculate the effect size, taking into account that values of approximately 0.20 indicate a small effect, values around 0.50 indicate a medium effect, and values of 0.80 or above indicate a large effect. Using Pearson’s correlation test, we examined the correlations between the study variables. Hierarchical linear regression analysis was performed to assess whether the correlated variables were significant predictors of insomnia, while controlling for other covariables. The prerequisite assumptions were verified prior to the regression analysis, namely homoscedasticity through residuals scatterplot, linearity through visual review of scatter plot pairs, and normality through visual examination of the predicted probability plots (Supplementary Materials). The Durbin-Watson test was used to determine the residuals’ independence. The absence of multicollinearity was ascertained using the Variance Inflation Factor (VIF) analysis. Using Hayes’ SPSS Process Macro Model 6 [60], we performed a serial mediation analysis to investigate the chain mediation pattern of cynical distrust, nightmare distress, anger, and insomnia. It should be emphasized that the serial mediation analysis used here is exploratory and associational in nature and that the proposed temporal ordering of variables is theoretically grounded but cannot be empirically confirmed with cross-sectional data. The reported regression coefficients were both unstandardized and standardized with their confidence intervals. 5000 bootstrap samples were used to determine the 95% confidence intervals. The absence of zero in the confidence intervals supports significant effects. SPSS software (Version 24.0) was used for the data analysis. The statistical significance threshold for all statistical analyses was set at *p* < 0.05 (two-tailed).

## 3. Results

### 3.1. General Characteristics of Participants and Scores on Outcome Variables

Common method bias testing was necessary because the study’s data were based on self-reports, and the Harman single-factor method test was used for this. The first common factor had an explanation rate of 28.340 percent, which was below the critical value of 50 percent, according to the results of the exploratory factor analysis. This suggests that there was no significant common method bias in this study.

The study involved 441 nurses in total (79 men and 362 women). This study sample and the entire population of nurses employed in Greece did not differ significantly in terms of gender, age, or years of professional experience [50]. In total, 62.1% of the nurses had symptoms of insomnia (AIS ≥ 6), 41.5% reported anger issues (DAR-5 ≥ 12), and 6.6 percent showed signs of nightmare distress (NDQ > 39). The mean value of cynical distrust in nurses (24.6 ± 6.24) was lower (*t*-test *p* < 0.05) than the value of 25.5 obtained in the general Greek population. Dividing the participants into quartiles (four groups based on CDS-8 scores) revealed that 20.9% of participants belonged to the highest quartile of the CDS-8 score distribution (CDS-8 > 29). The mean values and standard deviations of the study variables are displayed in Table 1.

In terms of gender, female nurses demonstrated higher mean scores on the DAR-5, CDS-8, NDQ and AIS compared to male nurses (*t*-test *p* < 0.05, 11.54 ± 3.92 vs. 9.84 ± 3.14, Hedges’ g: 0.448, *t*-test *p* < 0.05, 24.95 ± 6.16 vs. 22.88 ± 6.38, Hedges’ g: 0.333, *t*-test *p* < 0.05, 23.62 ± 9.38 vs. 20.2 ± 6.5, Hedges’ g: 0.382, and *t*-test *p* < 0.05, 7.6 ± 4.14 vs. 6.05 ± 3.95, Hedges’ g: 0.377, respectively, Table 1).

### 3.2. Correlations Among Continuous Variables

Anger, cynical distrust, nightmare distress, and insomnia showed significant positive correlations (Pearson Correlations *p* < 0.01, Table 2). Age was statistically negatively correlated with cynical distrust, nightmare distress, and insomnia (Pearson Correlations *p* < 0.05). Work experience and nightmare distress were also negatively correlated (Pearson Correlations *p* < 0.05).

### 3.3. Hierarchical Linear Regression Analysis

The prediction of AIS scores from participant general characteristics (age, gender, and work experience), DAR-5 scores, CDS-8 scores, and NDQ scores was assessed using a four-stage hierarchical linear regression analysis. The predictor variables age, gender (coded as 1 = male, 2 = female), and work experience were entered for the first block analysis. The results of the first block with age, gender, and work experience as predictor variables revealed a statistically significant model, F (3,433) = 4.225, *p* < 0.05, contributing 2.8% to the variation in insomnia. The second block analysis included the predictor variable DAR-5 scores, which made a significant contribution to the regression model (F (4,432) = 35.81, *p* < 0.001), explaining 22.1% of the variation in insomnia. An additional 1.5% of the variation in insomnia was explained by adding the CDS-8 scores variable at stage three. This change in R^2^ was significant, with F (5,431) = 30.928, *p* < 0.001. At the fourth stage, the NDQ scores variable was added, which further explained 10.2% of the variation in insomnia. This change in R^2^ was also significant, with F (6,430) = 41.311, *p* < 0.001. All the above results considered, insomnia was significantly predicted by the DAR-5, CDS-8, and NDQ scores. Notably, when the DAR-5, CDS-8, and NDQ variables were entered in the analysis, the significant effect from participant general characteristics (age, gender, and work experience) which were entered in the first block disappeared. Participants’ predicted insomnia was equal to −1.029 + 0.305(DAR-5) + 0.062(CDS-8) + 0.167(NDQ), (Table 3). Missing data were minimal (n = 4, <1%) and were handled via listwise deletion, resulting in an analytic sample of N = 437 for the hierarchical regression.

### 3.4. Mediation Analysis

**Figure 1:**
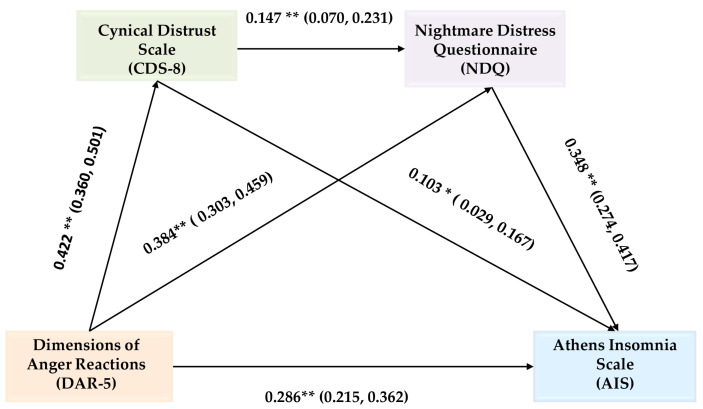
Chain Mediation model of Cynical distrust and Nightmare distress in the relationship between Anger and Insomnia. Note: * *p* < 0.05 or ** *p* < 0.01.

Subsequently, we investigated the possibility that the association between anger and insomnia could be mediated by cynical distrust and nightmare distress. The predictor variable in this analysis was DAR-5, the mediator variables were CDS-8 and NDQ, and the outcome variable was AIS. Age, gender, and work experience were not included in the model presented below because their effect was insignificant. Addressing potential concerns, we also conducted a mediation analysis that included age, gender, and years of work experience as covariates. The results remained largely unchanged, as shown in Supplementary Materials. The chain mediating role of cynical distrust and nightmare distress in the association between anger and insomnia was examined using Hayes’ SPSS Process Macro Model 6. 5000 bootstrap samples served as the basis for the analysis. Figure 1 shows the variables’ standardized coefficients along with their confidence intervals.

A Monte Carlo simulation was conducted to estimate the statistical power of the serial mediation model (PROCESS Model 6; Andrew F. Hayes) based on the observed parameter estimates and a sample size of N = 441. The analysis indicated that the power to detect the serial indirect effect (DAR-5 → CDS-8 → NDQ → AIS) was high (power ≈ 0.92), exceeding the conventional threshold of 0.80. These findings suggest that the present study was adequately powered to detect the hypothesized serial mediation effect.

According to the chain mediation analysis, the relationship between anger and insomnia is serially mediated by cynical distrust and nightmare distress. It was discovered that the combined indirect effect of nightmare distress and cynical distrust on insomnia was statistically significant [b = 0.2180, 95% C.I. (0.1560, 0.2850)]. Additionally, in the presence of the mediators, the direct effect of anger on insomnia was also found to be significant (b = 0.3086, *p* < 0.01). Consequently, the relationship between anger and insomnia is partially mediated by cynical distrust and nightmare distress. 41.39% of the variance in the AIS outcome variable can be explained by this model. Specifically, the following three pathways produced indirect effects that contributed to the overall mediating effect: (a) DAR-5 → CDS-8 → AIS, accounting for 8.62% of the total effect; (b) DAR-5 → NDQ → AIS, rendering 28.6% of the total effect; and (c) DAR-5 → CDS-8 → NDQ → AIS, constituting 4.17% of the total effect, (Table 4).

## 4. Discussion

This study investigated the interrelations among anger, cynical distrust, nightmare distress, and insomnia in a sample of Greek hospital nurses and, to our knowledge, is the first study to examine the serial mediating pattern that links these constructs together in an integrated manner. The results verified all four study hypotheses and revealed that anger correlates with insomnia through both direct and indirect sequential associations, with cynical distrust and nightmare distress serving as significant mediators. These results provide insight into the putative psychological processes that contribute to sleep disturbances among nursing staff.

A significant finding of this study is the high prevalence of insomnia symptoms (62.1%). These results are consistent with recent reports by studies in nursing populations, which found high rates of sleep disturbance [61,62]. Similar prevalence rates have been found in Greek nursing staff, where insomnia rates ranged around 60% [3,4,63], indicating that sleep disturbance continues to be a prevailing occupational health problem for this population group. The frequency of nightmare distress (6.6%) was also approximately equal to post-pandemic data from nursing staff in Greece [4], suggesting stability of nightmare distress over time. Taking them together, the findings underline that the sleep problems in nurses do not occur in isolation but are indicative of psychosocial stress responses linked to emotionally demanding clinical settings.

It is also important that 41.5% of nurses had clinically significant anger symptoms. This finding adds to the body of evidence, which suggests that nurses’ anger represents not so much an individual emotional phenomenon, but rather a reactive psychological response to chronic workplace adversity, such as moral distress, staff shortages, repeated exposure to suffering, workplace aggression, and lack of organizational support. Recent findings in Greek nurses have also shown strong links between anger and insomnia [3], meaning that anger may be an important transdiagnostic vulnerability factor for impaired sleep [64,65] in this population. In this sense, anger may be an externalization of unresolved feelings of helplessness, frustration and injustice.

The correlation analysis lent further support to the theoretical model we proposed. All of the principal study variables significantly and positively correlated with one another. The analysis indicated that the most robust association was between nightmare distress and insomnia, followed by anger and insomnia, suggesting that emotional hyperarousal and dysregulated nighttime emotional processing may trigger sleep disruption. These results are consistent with neurocognitive models of insomnia that place cognitive-emotional hyperarousal at the centre of the persistence of sleep problems, in addition to intrusive negative thoughts and physiological activation [66,67,68]. Also, the positive association between anger and cynical distrust supports research showing that hostility-related traits are linked with negative cognitive schemas and interpersonal distrust [69,70,71,72]. This positive association between anger and cynical distrust is in accordance with prior research showing that anger activates hostile attribution bias and negative interpretation of others’ intentions [73,74]. This finding lends credence to the idea that when nurses encountered stressful conditions and unfair treatment at work on a frequent basis, they may gradually develop enduring patterns of distrust.

The hierarchical regression analysis revealed that anger was the strongest initial psychological associated variable of insomnia, explaining 22.1% of the variance in AIS scores. This significant contribution demonstrates that anger is a clinically relevant correlate of sleep disturbance among nurses. While less pronounced, the contribution of cynical distrust to insomnia is also supported by literature investigating cynicism within the framework of burnout. Cynicism, as defined in burnout theory, has been linked with emotional exhaustion, depersonalization, and sleep disruption [75,76]. Nevertheless, the current study adds to this literature by examining cognitive distrust rather than cynicism and its consequences for sleep. Thus, the current study refines this understanding by focusing on cynical distrust as a cognitive schema, not merely an emotional or attitudinal component of burnout. Although prior studies have indicated that individuals with elevated hostility and distrust demonstrate heightened stress reactivity and adverse health outcomes [77,78], limited studies have directly investigated the association of cynical distrust with insomnia among nurses.

When cynical distrust and nightmare distress were incorporated into the model, the explained variance increased to 36.6%, signifying that the association between anger and insomnia is significantly enhanced by cognitive and nocturnal affective determinants. Notably, nightmare distress accounted for an additional 10.2% of explained variance, representing the largest incremental addition among the insomnia-associated variables. This finding aligns closely with prior evidence indicating that nightmare distress is significantly related to the severity of insomnia among nurses [4]. However, when compared to the extensive sleep literature, nightmare distress is still not sufficiently investigated in nursing samples [79]. Recent studies in nurses are mostly focused on insomnia, shift work and burnout [80,81,82] and little about dream-related phenomena. As a result of this study, nightmare distress emerges as an important sleep health factor among nurses that is often overlooked.

The findings on mediation analysis represent the most innovative contribution of this study. Past analyses have often restricted themselves to a single variable of interest, such as anger, cynical distrust, nightmare distress, or insomnia. In contrast, this study offers a theoretically plausible hypothesis for their interrelated and sequential associations. As posited, cynical distrust and nightmare distress significantly and sequentially mediated the relationship between anger and insomnia. The presence of a strong direct correlation accompanied by a significant indirect associational sequence implies partial serial mediation, indicating that anger relates to insomnia both independently and via downstream cognitive-emotional processes.

The first indirect associational sequence identified in this study starts with anger and extends to insomnia through cynical distrust, suggesting that anger may progressively intensify maladaptive interpersonal schemas characterized by suspicion, hostility, and negative attributions about the intentions of others [83]. In the context of hospitals, recurrent experiences of interpersonal conflict, perceived injustice, or organizational disrespect [84,85] may reinforce these distrustful cognitions that further increase cognitive hyperarousal and interfere with sleep initiation and maintenance. This interpretation is in line with the burnout literature, which suggests that cynicism and depersonalization correlate strongly with diminished sleep quality among nurses [86,87].

The second indirect associational sequence, which begins from anger, passes through nightmare distress and ends in insomnia, accounted for the largest proportion of the total indirect association (28.6%). This means that nightmare distress is the most important mediator in the model. This is theoretically plausible, as unresolved anger and persistent emotional activation may be integrated into dream content, thereby augmenting nightmare frequency, emotional intensity, and distress upon awakening. When nightmares become associated with fear of sleep, anticipatory anxiety and conditioned sleep avoidance may develop, thus perpetuating insomnia symptoms [88]. This putative process is especially important for nurses because they often have to deal with traumatic clinical events, suffering, and death.

Most importantly, the statistical significance of the serial associational sequence from anger to cynical distrust, then to nightmare distress, and finally to insomnia introduced a theoretically plausible hypothesis for the proposed chain mediation model. Pending further research investigation, this finding tentatively suggests that anger may initially modify daytime cognitive schemas, prompting nurses to adopt a more hostile and suspicious interpretative style, which may subsequently affect nocturnal emotional processing and nightmare distress, potentially resulting in insomnia [68,89,90]. Conceptually, this hypothetical serial associational sequence synthesizes daytime cognitive susceptibility with nocturnal emotional dysregulation, providing a more thorough elucidation of how occupational psychological strain possibly translates into sleep disorders. This integrated chain mediation pattern argues for the existence of a complex, multi-faceted psychological process.

The finding that cynical distrust associates with nightmare distress corroborates theoretical frameworks positing that maladaptive cognitive schemas (e.g., distrust, hostility) disrupt emotional processing during sleep [91,92]. This aligns with research demonstrating that individuals possessing negative cognitive styles are more susceptible to distressing dream content and increased emotional reactivity to nightmares [93]. Furthermore, the fact that the indirect associational sequence through nightmare distress is almost three times as strong (28.6%) as the correlation sequence through cynical distrust (8.62%) suggests that the emotional processing hypothesized pathways may serve as more dominant associated variables of insomnia than cognitive schemas, although both are significant. This complex relationship aligns with integrative models of insomnia that encompass both cognitive and affective elements.

Another clinically relevant finding was that female nurses had much higher scores on anger, cynical distrust, nightmare distress, and insomnia. Even though the multivariable models showed that gender-related outcomes after the introduction of psychological variables were no longer significant, these descriptive differences suggest that female nurses may have a heavier emotional load, which could be due to demands at work and in their personal lives. Previous studies on nurses have reported comparable gender differences in insomnia and nightmare distress [3,4,63,87]. The negative relationship between age and factors such as cynical distrust, nightmare distress, and insomnia corresponds with research indicating that younger or less experienced nurses may be more susceptible to psychological distress due to diminished coping abilities and professional adaptation [4,94,95,96]. Nonetheless, the existing literature on age-related outcomes is inconclusive, indicating the need for further studies [97].

The present findings hold important clinical and organizational implications. However, all implications statements are qualified as exploratory suggestions because they are based on cross-sectional associations, pending confirmation in intervention or longitudinal studies. Interventions designed to alleviate insomnia among nurses may extend beyond conventional sleep hygiene approaches and explore the emotional and cognitive aspects of disrupted sleep. Programs focusing on anger control [98], maladaptive distrust schemas, and nightmare-focused interventions, such as imagery rehearsal therapy [99] and cognitive behavioral therapy for insomnia [100], may warrant attention in future intervention research. At the organizational level, diminishing workplace injustice, enhancing supervisory support, mitigating exposure to violence, and bolstering psychological safety may indirectly enhance sleep outcomes by alleviating anger and distrust.

More specifically, the identification of cynical distrust as a mediator shows how important it is for leaders to create an environment of psychological safety, fairness, and trust within teams. Nursing managers may promote clear communication, fair workload distribution, supportive supervision, and embrace conflict-sensitive leadership behaviors [101,102,103]. Repeated experiences of perceived injustice, invalidation, or lack of recognition may harden hostile cognitive schemas that extend beyond the workplace, possibly linked to sleep-related emotional processing [104,105]. Policies that promote shared governance, participatory decision-making, and respectful interdisciplinary collaboration may consequently mitigate the emergence of cynical attitudes and their speculated subsequent ramifications.

It is important to recognize a number of limitations. First, the cross-sectional design prevents causal inferences, and while the serial mediation model is theoretically sound, definitive temporal ordering cannot be established. Subsequent research utilizing longitudinal or experimental methodologies is essential to elucidate causality and the persistence of these effects over time. Second, the research depended solely on self-report instruments, potentially leading to response biases including social desirability, recall bias, or subjective misinterpretation of questionnaire items. Due to the delicate nature of constructs such as anger and distrust, especially in professional settings where emotional expression may be limited, participants may have either underreported or overreported their experiences. Furthermore, employing self-reported sleep measures instead of objective assessments (e.g., actigraphy or polysomnography) may constrain the accuracy of the insomnia and nightmare-related outcomes. Third, the use of convenience sampling may limit generalizability, despite the sample’s satisfactory demographic representativeness; selection bias cannot be excluded. Nurses who were more stressed may have been either more motivated to take part or less likely to respond, which could have affected the observed prevalence rates. Fourth, the study was conducted exclusively among Greek hospital nurses, potentially limiting the generalizability of the findings to other cultural or healthcare settings. Cultural factors, organizational frameworks, and healthcare system attributes can affect the manifestation of anger, the emergence of distrust, and sleep-related consequences. Consequently, caution is warranted when generalizing these findings to nursing staff populations in different countries or care settings. Furthermore, online recruitment of participants may have introduced self-selection bias, and while the sample was compared to the national nursing population on key demographic variables (age, gender, years of experience) without yielding significant differences, generalizability to all Greek nurses should be interpreted with caution. Finally, although the study examined key psychological variables, potential confounding factors were not included in the analysis. Variables such as shift work patterns (e.g., night shifts, rotating schedules), department type (e.g., ICU, emergency, oncology), workload intensity, exposure to traumatic events, burnout, mental health history (such as anxiety, depression), prior sleep history, and coping strategies may have substantially influenced the observed associations. The omission of these factors may have led to an inadequate comprehension of the underlying associations and should be addressed in future studies with more comprehensive designs.

Despite these limitations, the study has significant strengths, including a substantial nursing sample, rigorous psychometric instruments, hierarchical regression modeling, and advanced serial mediation analysis employing bootstrapping techniques. Most importantly, it introduces a novel exploratory framework demonstrating that the associational sequence from anger to insomnia in nurses is not merely direct, but possibly unfolds through cynical distrust and nightmare distress. Future research studies could utilize longitudinal designs to ascertain causal relationships, employ intervention studies to evaluate the efficacy of concurrently addressing anger, cynical distrust, and nightmare distress, explore additional mediators or moderators, such as resilience, social support, or moral injury, broaden investigations to various cultural and healthcare contexts to improve generalizability, and conduct qualitative studies to gain deeper insights into how nurses perceive and interpret anger and distrust in their daily work environments.

As a theoretically plausible hypothesis rather than an empirical finding, individuals with an increased level of anger are more prone to develop cynical distrust towards others which might reinforce hostile cognitive attributions and also develop emotional hypervigilance. Longitudinal designs with ecological momentary assessment would be required to empirically test whether these cognitive-emotional processes during the day actually carry over into nocturnal emotional processing. The association of daytime psychological strain and nighttime distress [29] on the relationship between anger and insomnia relies on distrust-based cognitive vulnerability and emotional dysregulation associated with nightmares. Such findings hint that sleep problems in nursing staff may represent deeper emotional and cognitive processes related to workplace adversities. By addressing these hypothesized pathways, the sleep health of nurses can be enhanced, which will foster resilience, positively impact their psychological health and improve patient care.

## 5. Conclusions

The high prevalence of insomnia and anger symptoms observed in this study further underscores the considerable mental health challenges encountered by hospital nurses, particularly within demanding healthcare environments characterized by persistent stress, emotional exhaustion, and organizational pressures. Anger was found to be significantly correlated with insomnia, both directly and through its associations with cynical distrust and nightmare distress. The identification of an exploratory serial mediation model provides a more nuanced understanding of the psychological processes underlying sleep disturbances in this population. The proposed associational model requires longitudinal validation before any alleged mechanistic conclusions can be drawn. However, these findings likely suggest that insomnia in nurses may represent not only a sleep-related issue but also a broader manifestation of unresolved emotional and cognitive distress linked to workplace challenges. This advocates the need for comprehensive, multilevel interventions that tackle both emotional and cognitive aspects of distress, ultimately contributing to improved well-being among nurses.

## Figures and Tables

**Table 1:** Descriptive statistics of participants.

Participants		Age	Work Experience (In Years)	Dimensions of Anger Reactions	Cynical Distrust Scale	Nightmare Distress Questionnaire	Athens Insomnia Scale
Men N = 79	Mean	46.62 *	20.06	9.84 **	22.88 **	20.2 **	6.05 **
	S. D	10.56	11.61	3.14	6.38	6.5	3.95
Women N = 362	Mean	43.14 *	17.84	11.54 **	24.95 **	23.62 **	7.6 **
	S. D	10.83	11.91	3.92	6.16	9.38	4.14
Total N = 441	Mean	43.77	18.24	11.23	24.6	23.01	7.32
	S. D	10.86	11.88	3.84	6.24	9.02	4.14

* *t*-test *p* < 0.05 or ** *t*-test *p* < 0.01.

**Table 2:** Correlations among age, work experience (in years), DAR-5, CDS-8, NDQ and AIS.

Pearson Correlation N = 441		AGE	Work Experience (in Years)	DAR-5	CDS-8	NDQ
Work Experience (in Years)	r	0.868 **				
	*p*	0.000				
Dimensions of Anger Reactions (DAR-5)	r	−0.056	−0.040			
	*p*	0.238	0.406			
Cynical Distrust Scale (CDS-8)	r	−0.125 **	−0.082	0.424 **		
	*p*	0.009	0.085	0.000		
Nightmare Distress Questionnaire (NDQ)	r	−0.160 **	−0.170 **	0.442 **	0.296 **	
	*p*	0.001	0.000	0.000	0.000	
Athens Insomnia Scale (AIS)	r	−0.105 *	−0.077	0.485 **	0.335 **	0.520 **
	*p*	0.028	0.108	0.000	0.000	0.000

* *p* < 0.05 or ** *p* < 0.01.

**Table 3:** Summary of Hierarchical Regression Analysis for Variables predicting insomnia (AIS scores).

		Unstandardized Coefficients		Standardized Coefficients				
		B	Std. Error	Beta	t	Sig.	R Square	∆R^2^
Step 1	(constant)	6.538	1.563		4.183	0.000	0.028	0.028
	age	−0.049	0.036	−0.129	−1.343	0.180		
	gender	1.430	0.516	0.133	2.770	0.006		
	Work Experience	0.018	0.033	0.052	0.548	0.584		
Step 2	(constant)	2.031	1.433		1.418	0.157	0.249	0.221
	age	−0.042	0.032	−0.109	−1.294	0.196		
	gender	0.573	0.461	0.053	1.244	0.214		
	Work Experience	0.016	0.029	0.047	0.561	0.575		
	DAR-5	0.513	0.046	0.477	11.264	0.000 **		
Step 3	(constant)	0.285	1.537		0.186	0.853	0.264	0.015
	age	−0.032	0.032	−0.085	−1.015	0.311		
	gender	0.509	0.457	0.047	1.114	0.266		
	Work Experience	0.012	0.039	0.034	0.412	0.681		
	DAR-5	0.452	0.049	0.421	9.135	0.000 **		
	CDS-8	0.091	0.030	0.137	2.969	0.003 **		
Step 4	(constant)	−1.029	1.437		−0.716	0.475	0.366	0.102
	age	−0.035	0.030	−0.093	−1.190	0.235		
	gender	0.289	0.426	0.027	0.678	0.498		
	Work Experience	0.032	0.027	0.093	1.194	0.233		
	DAR-5	0.305	0.049	0.284	6.181	0.000 **		
	CDS-8	0.062	0.029	0.093	2.164	0.031 *		
	NDQ	0.167	0.020	0.364	8.299	0.000 **		

Correlations are statistically significant at the * *p* < 0.01 level or ** *p* < 0.05.

**Table 4:** Chain Mediation Analysis of Cynical distrust and Nightmare distress on Anger/Insomnia relationship.

Variable	b	SE	t	*p*	95% Confidence Interval	
					LLCI	ULCI
DAR-5 → CDS-8	0.6897	0.0705	9.7894	0.0000	0.5513	0.8282
DAR-5 → NDQ	0.9037	0.1106	8.1710	0.0000	0.6863	1.1211
CDS-8 → NDQ	0.1914	0.0681	2.8098	0.0052	0.0575	0.3253
DAR-5 → AIS	0.3086	0.0489	6.3058	0.0000	0.2124	0.4048
CDS-8 → AIS	0.0659	0.0283	2.3254	0.0205	0.0102	0.1215
NDQ →AIS	0.1666	0.0198	8.4269	0.0000	0.1278	0.2055
^(1)^ DAR-5 → CDS-8 → AIS	0.0454	0.0204			0.0076	0.0878
^(2)^ DAR-5 → NDQ → AIS	0.1506	0.0256			0.1031	0.2033
^(3)^ DAR-5 → CDS-8 → NDQ → AIS	0.0220	0.0085			0.0069	0.0399
Effects						
Direct	0.3086	0.0489	6.3058	0.0000	0.2124	0.4048
* Total Indirect	0.2180	0.0329			0.1560	0.2850
Total	0.5267	0.0449	11.7219	0.0000	0.4384	0.6150

Notes: Ind1: ^(1)^ DAR-5 → CDS-8 → AIS = DAR-5 → CDS-8 × CDS-8 → AIS, Ind2: ^(2)^ DAR-5 → NDQ → AIS = DAR-5 → NDQ × NDQ → AIS, Ind3: ^(3)^ DAR-5 → CDS-8 → NDQ → AIS = DAR-5 → CDS-8 × CDS-8 → NDQ × NDQ →AIS. * Total Indirect = Ind1 + Ind2 + Ind3, based on 5000 bootstrap samples.

## Data Availability

The data that support the findings of this study are available from the corresponding author, [A.T.], upon reasonable request.

## Supplementary Materials

The following supporting information can be downloaded at: https://www.mdpi.com/article/10.3390/jcm15103837/s1, Table S1: Normality of residuals; Table S2: Chain Mediation Analysis of Cynical distrust and Nightmare distress on Anger/ Insomnia relationship including Gender, Age and Work Experience (W.E.) in years, as covariates; Figure S1: Normality of residuals.
