# Supplementary material for: Anger, Cynical Distrust, Nightmare Distress and Insomnia Among Nursing Personnel
Source: J Clin Med. 2026 May 15;15(10):3837. doi: 10.3390/jcm15103837 (PMC13207250; doi:10.3390/jcm15103837)
Supplement: Supplementary file 1 [file jcm-15-03837-s001.zip › jcm-4296316-supplementary.pdf]

Table S1: Normality of residuals

One-Sample Kolmogorov-Smirnov Test

		Unstandardized Residual
N		437
Normal Parameters[a,b]	Mean	0E-7
	Std. Deviation	3,30215557
Most Extreme Differences	Absolute	,044
	Positive	,044
	Negative	−,028
Kolmogorov-Smirnov Z		,911
Asymp. Sig. (2-tailed)		,378

a. Test distribution is Normal.

b. Calculated from data.

Figure S1: Normality of residuals

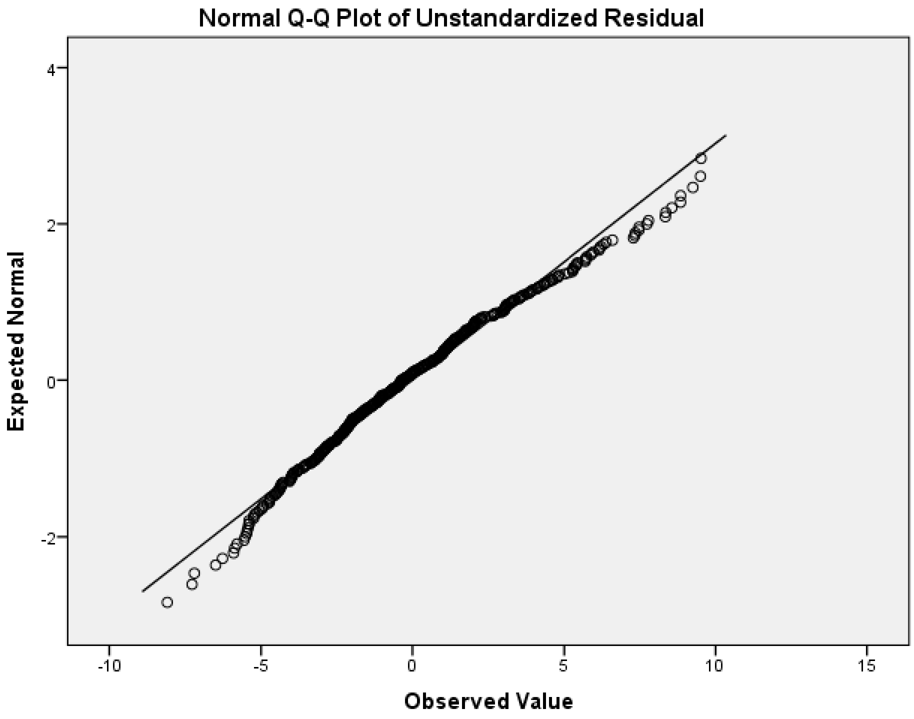

Table S2: Chain Mediation Analysis of Cynical distrust and Nightmare distress on Anger/ Insomnia relationship including Gender, Age and Work Experience (W.E.) in years, as covariates.

Variable	b	SE	t	p	95% Confidence Interval	
					LLCI	ULCI
DAR-5 → CDS-8	0.6685	0.0712	9.3920	0.0000	0.5286	0.8084
DAR-5 → NDQ	0.8831	0.1102	8.0118	0.0000	0.6664	1.0997
CDS-8 → NDQ	0.1722	0.0679	2.5364	0.0116	0.0388	0.3056
DAR-5 → AIS	0.3048	0.0493	6.1806	0.0000	0.2079	0.4017
CDS-8 → AIS	0.0618	0.0285	2.1640	0.0310	0.0057	0.1179
NDQ →AIS	0.1669	0.0201	8.2988	0.0000	0.1273	0.2064
(1) DAR-5 → CDS-8 → AIS	0.0413	0.0200			0.0035	0.0832
(2) DAR-5 → NDQ → AIS	0.1473	0.0253			0.1011	0.2013
(3) DAR-5 → CDS-8 → NDQ → AIS	0.0192	0.0080			0.0044	0.0361
Covariates						
Age → CDS-8	−0.1005	0.0502	−2.0038	0.0457	−0.1991	−0.0019
W.E. → CDS-8	0.0491	0.0457	1.0748	0.2831	−0.0407	0.1388
Gender → CDS-8	0.7040	0.7205	0.9770	0.3291	−0.7122	2.1202
Age →NDQ	0.0175	0.0711	0.2458	0.8060	−0.1223	0.1573
W.E → NDQ	−0.1220	0.0645	−1.8907	0.0593	−0.2488	0.0048
Gender → NDQ	1.3226	1.0179	1.2994	0.1945	−0.6780	3.3233
Age → AIS	−0.0353	0.0297	−1.1903	0.2346	−0.0937	0.0230
W.E. → AIS	0.0323	0.0270	1.1940	0.2331	−0.0209	0.0854
Gender → AIS	0.2887	0.4257	0.6782	0.4980	−0.5480	1.1255
Effects						
Direct	0.3048	0.0493	6.1806	0.0000	0.2079	0.4017
*Total Indirect	0.2079	0.0325			0.1485	0.2762
Total	0.5126	0.0455	11.2640	0.0000	0.4232	0.6021

Notes: Ind1: (1) DAR-5 → CDS-8 → AIS = DAR-5 → CDS-8 * CDS-8 → AIS, Ind2: (2) DAR-5 → NDQ → AIS = DAR-5 → NDQ * NDQ → AIS, Ind3: (3)DAR-5 → CDS-8 → NDQ → AIS = DAR-5 → CDS-8 * CDS-8 → NDQ * NDQ →AIS
*Total Indirect = Ind1+ Ind2+ Ind3, Based on 5,000 bootstrap samples.
